# *Populus × euramericana* Accumulates More Organic Pollutants (PAHs and PCBs), While *P. nigra* ‘Italica’ Absorbs More Heavy Metals

**DOI:** 10.3390/plants14101445

**Published:** 2025-05-12

**Authors:** Olivera Kalozi, Marko Kebert, Saša Orlović, Marko Ilić, Saša Kostić

**Affiliations:** 1 Faculty of Agriculture, University of Novi Sad, Trg Dostiteja Obradovića 8, 21000 Novi Sad, Serbia; olivera.kalozi@polj.edu.rs (O.K.); sasao@uns.ac.rs (S.O.); 2 Institute of Lowland Forestry and Environment, University of Novi Sad, Antona Čehova 13d, 21000 Novi Sad, Serbia; kebertmarko@gmail.com (M.K.); marko.ilic@uns.ac.rs (M.I.); 3 Laboratoire des Sciences du Climat et de l’Environnement, LSCE-IPSL (CEA-CNRS-UVSQ), 91190 Gif-sur-Yvette, France

**Keywords:** environmental pollution, biomonitoring, organic pollutants, heavy metals, poplar, phytoremediation, urban forestry

## Abstract

The phytoremediation capacity of three common poplar species, white poplar (*Populus alba* L.), Lombardy poplar (*Populus nigra* ’Italica’), and Euro-American hybrid poplar (*Populus × euramericana* (Dode) Guinier cl. I-214), grown in a middle-sized city with a continental climate in Serbia was analyzed. For this purpose, 15 polycyclic aromatic hydrocarbons (PAHs), 10 polychlorinated biphenyls (PCBs), and 6 heavy metals (HMs) were tracked in leaves and one-year-old branches. *P. × euramericana* showed the highest PAH uptake capacity, with concentrations of 821.40 ng g^−1^ dry weight (DW) and 453.64 ng g^−1^ DW in leaves and branches, respectively. Likewise, *P. euramericana* accumulated the highest levels of PCBs in leaves (364.53 ng g^−1^ DW). Additionally, *P. nigra* ‘Italica’ demonstrated the greatest accumulation potential for HMs, particularly zinc, with 310.10 µg g^−1^ DW in leaves. Leaves accumulated ~30% more pollutants compared with branches. Significant differences in pollutant uptake capacities were found among species and plant organs. These findings highlight the importance of species selection in phytoremediation and clarify the role of poplar species in accumulating pollutants to mitigate urban pollution. Finally, this study provides valuable insights for future phytoremediation strategies using poplars, especially in urban environments with similar conditions.

## 1. Introduction

Urban pollution is a critical issue in the entire Europe, with significant implications for public health and environmental quality. Among the various contaminants, polycyclic aromatic hydrocarbons (PAHs), polychlorinated biphenyls (PCBs), and heavy metals (HMs) are particularly concerning due to their persistence, toxicity, and potential for bioaccumulation [1]. The European Environment Agency [2] has been at the forefront of monitoring and assessing these pollutants in urban areas, providing valuable data and insights that inform policy and mitigation strategies [3]. The European Environment Agency [2] identifies numerous sources of environmental pollution, with the most significant contributors typically being industrial facilities, fossil fuel-based power plants, vehicle emissions, agricultural activities (such as pesticide and fertilizer use), and wastewater from various origins. Through comprehensive research and detailed reporting, the EEA [2] has highlighted the sources, distribution, and impact of PAHs, PCBs, and heavy metals in European cities.

Phytoremediation technology offers a promising solution by utilizing plants as ‘*biofilters*’ to accumulate and remove organic and/or inorganic contaminants from the soil, groundwater, and atmosphere, effectively retaining them in the roots, leaves, and branches [4,5,6,7]. Depending on site conditions and contaminant types, diverse techniques utilizing plants to mitigate environmental contamination have been developed, including phytoextraction, phytostabilization, phytodegradation, rhizofiltration, phytovolatilization [8,9]. Poplar and willow species, in particular, have demonstrated remarkable potential for phytoremediation due to their unique ability to accumulate HMs and other xenobiotics from whole environment (soil, water, and air) [10,11,12,13]. Poplars are particularly well suited for phytoremediation due to their rapid growth, high transpiration rates, ease of propagation, deep root systems, and strong capacity to accumulate and transport both essential and non-essential HMs into their above-ground parts but also due to the specific enzymes that can conjugate and detoxify various organic pollutants [14,15,16]. Glutathione-S-transferase, cytochrome P450, and various peroxidases are the most pivotal enzymes in PAH and PCB detoxification [17].

The extensive root systems of poplar trees further enhance their phytoremediation capabilities by effectively capturing pollutants from the soil and groundwater, thereby preventing their migration and further contamination of the surrounding environment [4,10,11,12,18]. This positions poplar species as pivotal contributors to sustainable environmental management practices, highlighting their potential as viable solutions for addressing pollution challenges and protecting ecosystem health, particularly in urban environments.

Two of the most notable xenobiotics in the environment, recognized as persistent organic pollutants (POPs), include polycyclic aromatic hydrocarbons (PAHs) and polychlorinated biphenyls (PCBs). These compounds pose substantial risks to ecosystems and living organisms, continuing to present serious threats to human health [19]. PAHs and PCBs can accumulate across environmental compartments, including soil, air, water, and biota [20], posing persistent risks to ecosystem health and biodiversity. PAHs are primarily generated from incomplete combustion of organic materials and are ubiquitous in urban settings, industrial areas, and contaminated sites [21]. These compounds are highly lipophilic and can accumulate into the living organisms, posing risks through various exposure routes such as inhalation, ingestion of contaminated food, and dermal contact [21]. PAHs are classified as human carcinogens compounds and have been linked to respiratory diseases, developmental abnormalities, and immune system disruption. In the environment, PAHs persist in soil and sediments, where they can adversely affect microbial and fungal communities and disrupt ecosystem functioning [22]. The main source of PAHs in urban environments is derived from anthropogenic activities, including vehicle emissions [23,24,25,26], domestic heating [27,28], or industrial processes [29,30,31]. They are a major concern because of their high concentration in most urban areas, their wide distribution and accumulation, and their toxic effects on living organisms.

The US Environmental Protection Agency [32] and the EU have identified 16 priority PAHs that are of particular concern as environmental pollutants [33,34]. Due to their persistence in the environment and ability to accumulate in organisms, these 16 PAHs pose serious risks to human health and well-being as they are well known to be carcinogenic, mutagenic, and teratogenic [35]. Furthermore, the International Agency for Research on Cancer (IARC) classifies certain PAHs into four groups based on their carcinogenic potential (CPAHs). These groups include benzo[a]pyrene (BaP) categorized as Group 1 carcinogens, dibenzo[a,h]anthracene (DA) is placed in Group 2A, and chrysene (CRY), benzo[b]fluoranthene (BbF), benzo[k]fluoranthene (BkF), and indeno[1,2,3-cd]pyrene (IcdP) are in Group 2B. In total, 9 out of the 16 priority PAHs are classified under Group 3. Therefore, the monitoring and regulation of these compounds are essential to mitigate their adverse effects on both environmental and human health.

PCBs, once widely used in industrial applications [36,37], are persistent and bioaccumulative compounds. Despite bans on their production, PCBs continue to pose risks due to their stability and presence in the environment [38]. They are known to cause neurotoxicity, impair reproductive health, and disrupt endocrine systems in humans [39]. PCBs also bioaccumulate in the food chain, leading to higher concentrations in predators such as fish and mammals, which can result in chronic exposure to humans consuming contaminated seafood [40]. Environmental impacts of PCBs include their persistence in soil and sediments, where they can lead to long-term contamination and adverse effects on wildlife populations, including reproductive impairments and immune system suppression [41].

Heavy metals in urban environments pose significant environmental and public health concerns due to their persistence, toxicity, and widespread sources. These HMs, including lead (Pb), cadmium (Cd), mercury (Hg), arsenic (As), and chromium (Cr), originate from various anthropogenic activities such as industrial processes, vehicular emissions, mining, and improper waste disposal [20,42]. In urban settings, concentrations of HMs are often elevated in soils, sediments, air, and water and have detrimental effects on ecosystems, affecting soil quality, plant growth, and microbial communities. The efficiency of poplars in the phytoextraction of various HMs varies depending on the species, type of metal, and concentrations present in the soil and atmosphere. The best-understood cysteine-rich proteins and metal-binding ligands involved in heavy metal detoxification in plants are phytochelatins and metallothioneins [43,44].

The main objective of this study was to evaluate the phytoremediation potential of three common poplar species—white poplar (*Populus alba* L.), Lombardy poplar (*Populus nigra* ‘Italica’ Du Roi), and Euro-American hybrid poplar (*Populus × euramericana* (Dode) Guinier cl. I-214)—in urban environments within a moderate continental climate. This study focused on quantifying the uptake of (I) polycyclic aromatic hydrocarbons (PAHs), (II) heavy metals (HMs), and (III) polychlorinated biphenyls (PCBs) by leaves and one-year-old branches. The study also aimed to elucidate the relationships between absorbed environmental pollutants and their distribution among different plant parts, emphasizing compounds with carcinogenic potential. Understanding the distinct capabilities of each species in absorbing and sequestering targeted xenobiotics within their vegetative organs is critical for assessing variations in uptake capacities.

## 2. Results and Discussion

### 2.1. Polycyclic Aromatic Hydrocarbon (PAH) Phytoaccumulation

**Figure 1:**
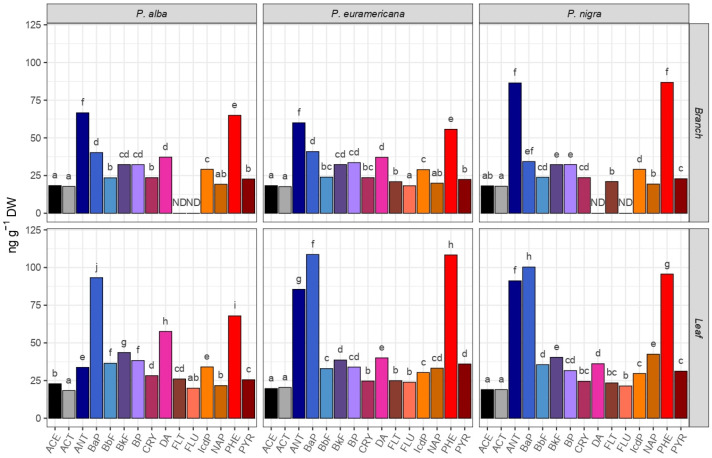
Phytoaccumulations of 15 polycyclic aromatic hydrocarbons (PAHs) detected separately in 1-year-old branches and leaves in *Populus alba*, *P. × euramericana* cl. I-214, and *P. nigra* ‘Italica’. Different small letters indicate statistically significant differences among different PAHs based on Tukey’s honestly significant difference (HSD) post hoc test for *p* ≤ 0.05. Data represent a mean value expressed in ng g^−1^ DW. Abbreviation: acenaphtylene (ACE), acenaphthene (ACT), anthracene (ANT), benzo(a)pyrene (BaP), benzo(b)fluoranthene (BbF), benzo(k)fluoranthene (BkF), benzo(g,h,i)perylene (BP), crysene (CRY), dibenzo(a,h)anthracene (DA), fluoranthene (FLT), fluorene (FLU), indeno(1,2,3-cd)pyrene (IcdP), naphthalene (NAP), non-detected (ND), phenanthrene (PHE), and pyrene (PYR).

Out of the three analyzed poplar species, *P. × euramericana* cl. I-214 presented the highest potential for PAH uptake—based on the Ʃ_15_ PAH concentration (821.40 ng g^−1^ DW absorbed PAH in leaves; 453.64 ng g^−1^ DW in branches), while the lowest capacity for PAH uptake (597.68 ng g^−1^ DW in leaves and 397.74 ng g^−1^ DW in branches) was observed in *P. alba* (Figure 1). This aligns with findings by Rodriguez et al. [45], who also reported significant PAH accumulation in hybrid poplars (*Populus hybridus*), specifically noting high concentrations of PAHs in their leaves. On the other hand, *P. nigra* ‘Italica’ and *P. alba* branches did not absorb FLU, while *P. alba*, in addition to FLU, also did not absorb FLT, and *P. nigra* ‘Italica’ did not absorb DA. *P. × euramericana* cl. I-214 branches absorbed the least amount of ACT (60.01 ng g^−1^ DW), and its leaves absorbed the least amount of ACE (19.71 ng g^−1^ DW), while the leaves of the other two species (*P. alba* and *P. nigra* ‘Italica’) absorbed the least amounts of ACT (18.34 and 19.00 ng g^−1^ DW, respectively). The three most commonly absorbed PAHs were ANT, BaP, and PHE in both (leaves and branches). The highest concentrations of ANT were observed in the branches of *P. alba* and *P. × euramericana* cl. I-214 (66.61 and 60.01 ng g^−1^ DW, respectively). Likewise, BaP was absorbed in the highest concentrations by the leaves of *P. alba* and *P. nigra* ‘Italica’ (123.29 and 100.32 ng g^−1^ DW, respectively), while the branches of *P. nigra* ‘Italica’ and the leaves of *P. × euramericana* cl. I-214 absorbed the highest amounts of PHE (86.78 and 178.37 ng g^−1^ DW, respectively).

Out of a total of 15 quantified PAHs, 6 have been classified as carcinogenic (CPAHs)—CRY, BbF, BkF, BaP, IcdP, and DA—following the Provisional Guidance for Quantitative Risk Assessment of Polycyclic Aromatic Hydrocarbons [32]. The highest amount of CPAHs was absorbed by the leaves of *P. alba* (323.24 ng g^−1^ DW, representing 54.1% of the total PAHs absorbed), with BaP being the most dominant among the CPAHs (123.29 ng g^−1^ DW). A study by Widdowson et al. [46] conducted a long-term remediation assessment using hybrid poplars at a creosote-contaminated site, observing a reduction in the area extent of the PAH plume, with significant declines in PAH concentrations in groundwater over 7 years, particularly for NAP and three-ring PAHs like ACE and ACT. However, four-ring PAHs persisted in the lower depths of the saturated zone. Our study, however, demonstrated a more comprehensive absorption of various PAHs, including more persistent four-ring PAHs such as CRY and BbF, particularly by *P. alba*. This suggests that *P. alba* may have a higher potential for the remediation of these more recalcitrant PAH compounds compared with other species.

Following in CPAH quantity are the leaves of *P. alba* and *P. nigra* ‘Italica’, branches of *P. × euramericana* cl. I-214, leaves of *P. × euramericana* cl. I-214, and branches of *P. nigra* ‘Italica’ (with CPAHs representing 41.9%, 41.5%, 41.2%, 37.2%, and 29.2% of the total PAHs absorbed by each, respectively).

Based on two-way ANOVA, the organ was the dependent variable that showed a statistically significant difference (*p* < 0.05) in the accumulation of the analyzed PAHs (Table 1). Significant differences among different species were observed for 6 out of the 15 analyzed PAHs (NAP, ACT, FLU, PHE, ANT, and DA) at the *p* < 0.05 level. Strong interaction effects among species and organs were observed for PHE (*F* = 22.739, *p* = 8.28 × 10^−5^), ANT (*F* = 21.239, *p* = 1.14 × 10^−4^), and DA (*F* = 9.430, *p* = 0.012), indicating that the organs’ impact on the accumulation of these PAHs varies significantly among different species. Conversely, PAHs such as ANT (*F* = 6.378, *p* = 0.270), FLT (*F* = 2.715, *p* = 0.130), CRY (*F* = 2.645, *p* = 0.132), IcdP (*F* = 0.787, *p* = 0.398), and BP (*F* = 0.552, *p* = 0.591) did not exhibit significant organ-specific accumulation or interaction effects, suggesting a more uniform distribution across different organs and species.

Numerous studies have shown that the use of woody plant species in urban areas can significantly contribute to remediation, particularly in the degradation and removal of xenobiotics from the surrounding environment. For instance, Kostić et al. [47] utilized *Platanus x acerifolia* ((Aiton) Willd.), *Tilia grandifolia* (Ehrh.), and *Celtis australis* (L.) for the phytoremediation of PAHs in urban environments, and demonstrated that *C. australis* accumulated the highest concentrations of PAHs in its leaves and branches. Likewise, Liang et al. [48] employed 12 different plant species along an urbanization gradient in Shanghai, identifying *Pittosporum tobira* ((Thunb) Aiton.), *Ginkgo biloba* (L.), and *Platanus x acerifolia* ((Aiton) Willd.) as the most efficient species for adsorbing PAHs. Gréau et al. [49] investigated the response of poplar and associated fungal endophytic communities to PHE contamination gradient, discovering that poplar growth was significantly reduced when PHE concentrations exceeded 400 mg kg^−1^. Additionally, the study observed changes in the composition of the fungal community, suggesting a toxic effect of PHE on certain fungal taxa. These findings complement our research by highlighting the broader ecological impact of PAH contamination on both plants and their associated microbial communities. Additionally, Wittig et al. [50] examined the exposure of *Populus nigra* L. cv. Loenen to various PAHs and its effect on growth and water balance. They found that PAH exposure significantly affected root mass, nutrient solution uptake, and transpiration rates, with FLT showing the highest phytotoxic potential. These physiological changes underline the importance of understanding the specific responses of different poplar species to various PAHs, which can inform the selection of the most suitable species for phytoremediation purposes. Overall, these findings highlight both the unique capabilities and challenges associated with different poplar species in accumulating and remediating various PAHs. By comparing our results with those of other studies, it is evident that poplars, especially *P. alba*, have substantial potential for the phytoremediation of persistent and carcinogenic PAHs in urban environments.

### 2.2. PCB Phytoaccumulation

**Figure 2:**
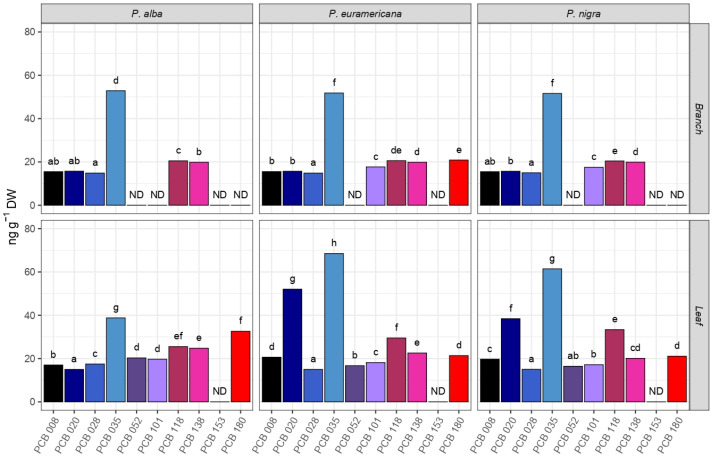
Phytoaccumulation of 10 polycyclic biphenyls (PCB: 8, 20, 28, 35, 52, 101, 118, 153, 138, and 180) separately in 1-year-old branches and leaves in *Populus alba*, *P. × euramericana* cl. I-214, and *P. nigra* ‘Italica’. Different small letters indicate statistically significant differences among different PCBs based on Tukey’s honestly significant difference (HSD) post hoc test for *p* ≤ 0.05, while ND is not detected. Data represent a mean value expressed in ng g^−1^ DW.

Following Ʃ_10_ PCB concentration, the leaves of *P. × euramericana* cl. I-214 exhibited the highest capacity for PCB accumulation (364.53 ng g^−1^ DW), while the lowest foliar concentration was observed in *P. alba* (182.64 ng g^−1^ DW). The highest concentration of PCBs was also observed in the branches of *P. × euramericana* cl. I-214 (137.52 ng g^−1^ DW), while the lowest concentration was found in *P. nigra* ‘Italica’ (108.42 ng g^−1^ DW). PCB 52 was not identified in the branches of all three poplar species, while PCB 180 was not identified in the branches of *P. alba* and *P. nigra* ‘Italica’. Likewise, PCB 101 was also not identified in the leaves of *P. alba*, while PCB 118 was not present in the branches of *P. × euramericana* cl. I-214 (Figure 2). These findings align with those of Liu and Schnoor [51], who reported that hybrid poplars effectively accumulated lesser-chlorinated PCBs, such as PCB 28 and PCB 52, predominantly in the root systems, with some translocation to the stems but not beyond. Similarly, our study did not detect PCB 52 in the branches, whereas *P. × euramericana* cl. I-214 demonstrated a more extensive distribution of PCBs within the leaves and branches, indicating a higher potential for overall PCB remediation in urban settings. Zhai et al. [52] found that all above-ground poplar organs could biotransform chiral PCBs, with significant accumulation in the root and bark. However, their study primarily focused on the biotransformation capabilities rather than the total accumulation. This further emphasizes the importance of understanding both the accumulation and transformation processes to fully utilize poplars in phytoremediation.

All observed poplar species absorbed the highest amounts of PCB 35, ranging from 21.23% to 47.59% of the total amount of absorbed PCBs. Statistically significant differences were observed among different species in the accumulation of several analyzed PCBs (PCB 8, PCB 20, PCB 35, and PCB 180; *p* < 0.05; Table 1). The same pattern is evident among organs, with a difference noted in PCB 118, where the *p*-value is 0.016. When analyzing the species–organ interaction effect on PCB distribution, a statistically significant difference is only observed in the accumulation of PCB 8 and PCB 35.

Results of two-way ANOVA showed that the organ is a dependent variable showing a statistically significant difference (*p* < 0.05) in the accumulation of several analyzed PCBs (PCB 8, PCB 20, PCB 35, PCB 118, and PCB 180). The organ did not significantly affect the accumulation of PCB 28, PCB 101, and PCB 138 (Table 1). Significant differences between different species were observed for PCB 8, PCB 20, PCB 35, and PCB 180 at the *p* < 0.05 level. Additionally, the F-test for PCB 35 indicates a strong interaction effect between species and organ on their distribution.

In their study, Ancona et al. [11] noted that the bioaccumulation factor values for PCBs in poplar roots and leaves were relatively low, indicating limited translocation from soil to plant tissues. Our findings in leaves and branches align with the observed limited translocation of PCBs to other parts of the plants. Furthermore, Ancona et al. [53] demonstrated that the Monviso clone of poplar was particularly effective in absorbing more persistent four-ring PCBs, suggesting a higher potential for the remediation of these more recalcitrant compounds compared with other species. Our study, which showed significant potential of *P. × euramericana* cl. I-214 for PCB phytoremediation, indicates that different poplar species may have complementary roles in the remediation process. These findings underscore the substantial potential of poplars for the phytoremediation of persistent PCBs in urban environments. Poplars can play a crucial role in mitigating PCB pollution, improving environmental health, and contributing to sustainable urban ecosystem management [51,53].

### 2.3. Heavy Metal Phytoaccumulation

**Figure 3:**
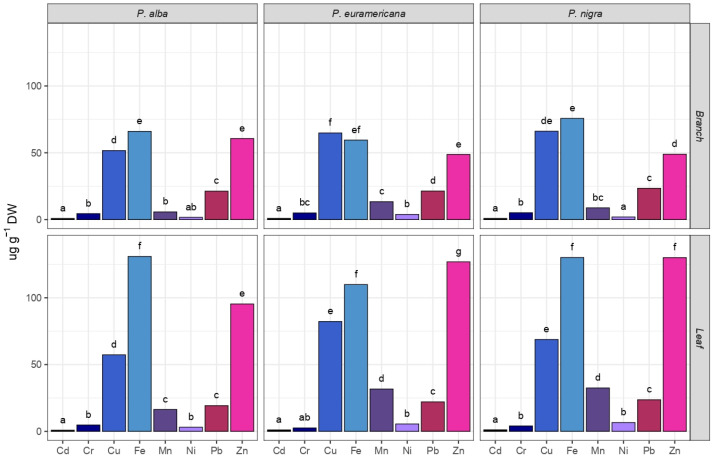
Phytoaccumulation of eight heavy metals (Cd, Cr, Cu, Fe, Mn, Ni, Pb, and Zn) in leaves and 1-year-old branches in *Populus alba*, *P. × euramericana* cl. I-214, and *P. nigra* ‘Italica’. Different small letters indicate statistically significant differences among different HMs based on Tukey’s honestly significant difference (HSD) post hoc test for *p* ≤ 0.05). Data represent a mean value expressed in µg g^−1^ DW.

Based on the Ʃ_8_ HM concentration in both leaves and branches, *P. nigra* ‘Italica’ showed the highest accumulation potential (664.02 and 231.05 µg g^−1^ DW, respectively), opposite to *P. alba* (378.02 and 212.35 µg g^−1^ DW, respectively; see Figure 3.). The least abundantly accumulated heavy metal in all the three observed poplar species was Cd, ranging from 0.86 to 1.16 µg g^−1^ DW. The most abundantly accumulated element was Zn, which was absorbed by the leaves of *P. nigra* ‘Italica’ at a concentration of 310.10 µg g^−1^ DW. Zn was also most prominently absorbed by the leaves of *P. × euramericana* cl. I-214 (116.94 µg g^−1^ DW). Baldantoni et al. [54] observed that Cd and Zn were predominantly accumulated in the leaves of the poplar clones, while Cu, Fe, and Pb tended to accumulate more in the roots. This is consistent with our findings for Zn, which was also most prominently absorbed by the leaves of *P. nigra* ‘Italica’. However, in our study, Cd was the least accumulated HM across all the three poplar species, suggesting possible variations in soil composition, environmental conditions, or specific genetic differences among the poplar clones that were used. While Jakovljević et al. [10] observed that *P. nigra* could adapt to prolonged Cd exposure, resulting in high total Cd accumulation in aerial parts due to substantial biomass production, our study found lower overall Cd accumulation. However, *P. nigra* ‘Italica’ did exhibit the highest Cd uptake among the three species studied, suggesting some consistency with the findings of Jakovljević et al. [10] regarding this species’ relative efficiency in Cd accumulation despite the generally low Cd levels observed in our study. This indicates that while *P. nigra* ‘Italica’ has potential for Cd phytoremediation, environmental and soil conditions play a crucial role in determining the overall uptake levels. Further, in our study, Fe was the heavy metal most prominently absorbed by the leaves of *P. alba*, the branches of *P. nigra* ‘Italica’, and *P. alba* (180.95, 75.72, and 66.01 µg g^−1^ DW, respectively). A statistically significant difference was observed among different species in the accumulation of almost all HMs, except for Cr and Pb. The same trend is observable among organs, albeit with the accumulation of Cr showing statistical significance (Table 1). The accumulation of Pb has shown statistical significance, and when considering the interaction among species and organs, along with Fe and Ni.

Two-way ANOVA revealed that the organ was the dependent variable demonstrating a statistically significant difference (*p* < 0.05) in the accumulation of almost all analyzed HMs, with the exception of Pb (*p* = 0.731), which was also not significant for species (*p* = 0.088) and species x organ interaction (*p* = 0.574). Cr showed a trend towards significance for species (*p* = 0.059), while the species x organ interactions were not significant for Fe (*p* = 0.170) and Ni (*p* = 0.200). The highest *F*-value observed was for Mn in the organ category (*F* = 132.994, *p* = 7.52 × 10^−8^), demonstrating a highly significant difference in Mn accumulation between different organs. Cu also showed a strong effect with an *F*-value of 38.201 (*p* = 6.26 × 10^−6^) for species, indicating significant differences in Cu accumulation among species. Additionally, the organ effect for Fe was notable with an *F*-value of 40.468 (*p* = 3.6 × 10^−5^), highlighting significant differences in Fe accumulation between organs. These high F-values suggest that Mn, Cu, and Fe are the most influenced by organ and species differences.

The phytoremediation potential of various poplar species for HM accumulation shows significant variability based on genetic limitations and environmental factors. In our study, *P. nigra* ‘Italica’ exhibited the highest HM accumulation in leaves and branches, with values reaching 664.02 and 231.05 µg g^−1^ DW, respectively. When comparing the results obtained for *P. × euramericana* cl. I-214 with those reported by Kovačević et al. [55], certain similarities and differences emerge. In both studies, Fe was among the most accumulated metal, while Cd showed the lowest uptake. However, Zn concentrations were notably higher in our study compared with those in their findings, which may reflect differences in environmental conditions. Kebert et al. [12] observed that *Populus deltoides* (W.Bartram ex Marshall) exposed to high levels of Ni and Cd showed increased antioxidant enzyme activities and phenolic compound accumulation, which are indicative of greater tolerance to heavy metal stress. This finding supports the broader concept that poplar species have the capacity to develop physiological mechanisms for heavy metal tolerance, a notion that aligns with our observations of poplar species’ potential for enhanced phytoremediation efficiency. For example, Pilipović et al. [56] reported that various poplar clones grown in heavy metal-polluted soils showed significant differences in metal accumulation, while some clones demonstrated higher tolerance and accumulation capacities. This variability among clones supports the reliability for selecting specific genotypes from a gene pool for targeted phytoremediation pollutants. The choice of species and clones should be guided by the specific contaminants and environmental conditions of the site, ensuring optimal uptake and stabilization of HM. In addition to their remediation potential, poplars are also recognized as valuable bioenergy crops, as their ability to tolerate and accumulate HMs on contaminated sites enhances their role in both environmental restoration and sustainable biomass production [7]. This comprehensive approach improves the potential for successful remediation and environmental protection.

### 2.4. Species and Organ Capacity of PAH, PCB, and HM Uptake

**Figure 4:**
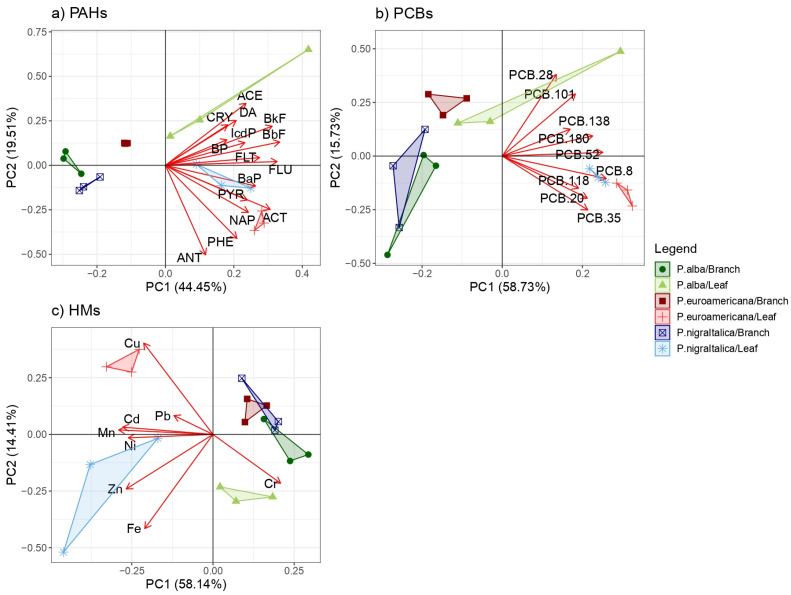
Principal component analysis of (**a**) polycyclic aromatic hydrocarbons (PAHs), (**b**) polycyclic biphenyls (PCBs), and (**c**) heavy metals (HMs) accumulated by leaves and 1-year-old branches of *P. alba* (green marks), *P. × euramericana* cl. I-214 (red marks), and *P. nigra* ‘Italica’ (blue marks) for the first two principal components (PCs). Abbreviation: acenaphtylene (ACE), acenaphthene (ACT), anthracene (ANT), benzo(a)pyrene (BaP), benzo(b)fluoranthene (BbF), benzo(k)fluoranthene (BkF), benzo(g,h,i)perylene (BP), crysene (CRY), dibenzo(a,h)anthracene (DA), fluoranthene (FLT), fluorene (FLU), indeno(1,2,3-cd)pyrene (IcdP), naphthalene (NAP), phenanthrene (PHE), and pyrene (PYR).

A predominant impact of species was also evident in the PCA graphs (Figure 4) for both leaf and branch samples. Cumulatively, PC1 and PC2 together described the entire distribution/variability of PAH, PCB, and HM concentrations throughout both leaf and branch samples, 63.69%, 74.46%, and 72.55%, respectively.

The PCA analysis of the 15 target PAHs indicated that all three analyzed species exhibited equal deviations from each other, with ACE, DA, and CRY concentrations notably distinguishing the leaf samples of *P. alba*, while for *P. nigra* ‘Italica’, BaP and PYR stood out, and for *P. × euramericana* cl. I-214, it was ACT and NAP (Figure 4). The smallest deviations between the analyzed specimens in the group were noted for the branches of all the three species.

For PCBs, a similar pattern was revealed by the PCA analysis, indicating that all the three analyzed species exhibited equal deviations from each other. The smallest deviations among the analyzed specimens were also noted for the branches of all the three species, with concentrations of PCB 28 and PCB 101 notably distinguishing the leaf samples of *P. alba*, while for the other two species, it was PCB 8 (Figure 4). The PCA analysis for HMs also showed the smallest deviation for the branches of all the three species. Leaf samples of *P. alba* exhibited higher concentrations of Cr, *P. × euramericana* cl. I-214 showed elevated Cu levels, and *P. nigra* ‘Italica’ displayed increased concentrations of Ni and Zn (Figure 4).

**Figure 5:**
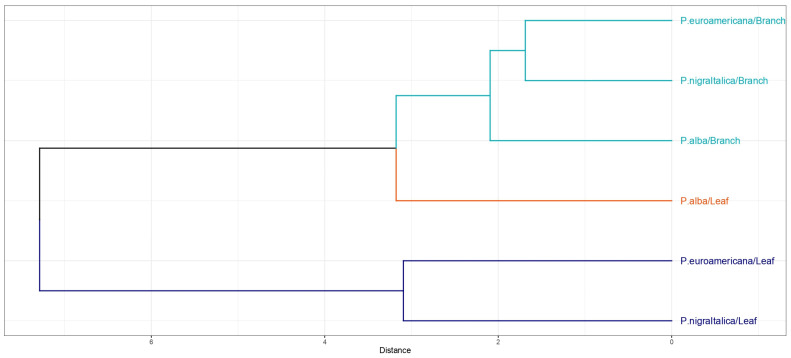
Dendrogram of hierarchical cluster analysis for all analyzed 33 xenobiotics (15 PAHs, 10 PCBs, and 8 HMs), grouped by species and organ into three cluster defined by color.

**Figure 6:**
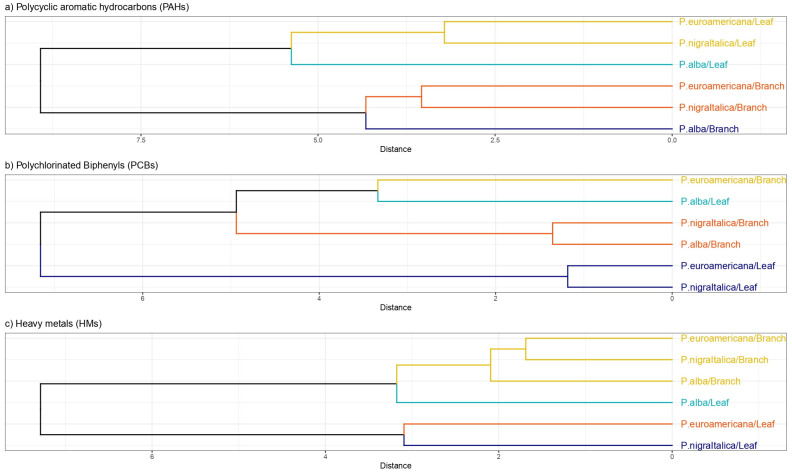
Hierarchical cluster analysis diagram of the accumulated (**a**) PAHs, (**b**) PCBs, and (**c**) HMs in *Populus alba*, *P*. *×* euramericana cl. I-214, *P. nigra* ‘Italica’ leaves and one-year-old branches into three cluster defined by color.

Furthermore, dendrograms of the cluster analysis (Figure 5 and Figure 6) visually illustrated the xenobiotics’ differences in uptake by species and their organs. The dendrogram of the cluster analysis of all the targeted xenobiotics differentiates the sample into two sub-clusters at a relatively smaller distance, which further divide into several sub-clusters at smaller distances (Figure 5). Samples of *P. × euramericana* cl. I-214 and *P. nigra* ‘Italica’ branches cluster at the smallest distance, indicating high similarity in xenobiotic accumulation between these two tree species. *P. alba* branches are generated at a slightly larger distance. Samples of *P. × euramericana* cl. I-214 and *P. nigra* ‘Italica’ leaves constitute the second sub-cluster.

If we observe the dendrograms of hierarchical cluster analysis separately for each absorbed xenobiotic, a similar pattern can be noticed. By concurrently interpreting three dendrograms, it can be concluded that the samples cluster at relatively larger distances. For accumulated PAHs (Figure 6a) and PCBs (Figure 6b), leaves of *P. × euramericana* cl. I-214 and *P. nigra* ‘Italica’ cluster at the smallest distance, while for the absorbed HM (Figure 6c), it involves branch samples of the same species. The dendrogram for PAHs (Figure 6a) shows a clear clustering pattern where the leaves and branches of the same species tend to group together, with smaller variation in PAH accumulation within the branches compared with the leaves, as evidenced by the tighter sub-clusters at shorter distances. The dendrogram that represents PCB data (Figure 6b) shows distinct clustering between leaves and branches. Shorter distances within the branch cluster indicate less variation in PCB accumulation compared with the leaves, which indicate wider variations. Lastly, the dendrogram for HMs (Figure 6c) shows clear clustering between leaves and branches, with a slightly different arrangement compared with PAHs (Figure 6a), suggesting different accumulation patterns for HMs.

### 2.5. Poplars as a PAH, PCB, and HM Remediation Tool in an Urban Environment

Environmental contamination by potentially toxic elements is a critical global issue, especially common in urban settings [57,58]. As previously mentioned, the main sources of environmental pollution are industrial facilities, power plants that use fossil fuels, vehicle emissions, agricultural practices, and wastewater from various sources [2,59]. Heavily polluted urban areas pose a significant challenge in eliminating sources of environmental contaminants [47,60]. Phytoremediation has shown significant potential in this regard, particularly for contaminants from traffic emissions, such as PAHs, PCBs, and HMs. Alexandrino et al. [24] highlighted that PAH concentrations in plant leaves varied significantly based on proximity to traffic and other emission sources, demonstrating a higher accumulation in areas with heavy vehicular activity. Certain species, such as *S. nigra* (L.) and *A. melanoxylon* (R.Br.), showed distinct patterns of PAH absorption, with specific PAHs like NAP and BaP being strongly associated with vehicular emissions. PAH concentrations are significantly higher in urban areas compared with non-urban areas, with traffic identified as the dominant source [47]. High-molecular-weight PAHs, typically associated with vehicle emissions, are more abundant in particulate matter collected from urban tunnels, reinforcing the role of traffic in PAH pollution [25]. Prasse et al. [61] noted that while vehicular exhausts are a minor source, the contribution of PCBs from traffic-related sources, such as lubricating oils, cannot be entirely dismissed. Trees in urban settings accumulate higher levels of heavy metals, with traffic being a primary contributor [62]. Significant levels of heavy metals in roadside soils and vegetation due to traffic emphasize the importance of phytoremediation in mitigating these pollutants [63]. The distribution and concentrations of xenobiotics observed in this study align with patterns typically associated with urban areas characterized by significant traffic density and industrial activities. As a relatively major urban center, Novi Sad experiences high levels of vehicular movement and population flux, both of which are recognized contributors to urban pollution [23,24,25,26]. These factors likely play a key role in the pollutant loads identified, reflecting trends observed in similarly sized cities in regions with comparable urban and environmental characteristics. Such findings underline the importance of traffic and industrial emissions as focal points for pollution mitigation in urban phytoremediation strategies.

Poplars are effective at absorbing a variety of xenobiotics, including both organic contaminants and heavy metals through their extensive root systems and high biomass production [15]. Capuana [64] emphasized the potential of poplar species in urban areas for phytoremediation due to their adaptability, fast growth, and greater biomass compared with herbaceous species. On top of that, he pointed out that the rooting depth of the *Populus* species and ease of propagation make them particularly suitable for remediation. Padoan et al. [57] demonstrated that short rotation coppice phytoremediation with poplar and *Salix* species, particularly clones P1, S1, and S3, effectively reduced bioavailable Zn in soil over 2 years. Their study highlighted the effectiveness of poplar species for urban soil remediation, emphasizing the need for extended durations and enhanced biomass yield through phytomanagement to achieve significant remediation. Pilipović et al. [65] found significant differences in metal accumulation among poplar clones in polluted soils, with some showing higher tolerance and accumulation capacities, supporting a targeted genotype selection to enhance phytoremediation effectiveness. Zalesny et al. [66] showed that poplars can mitigate soil and water contamination in urban environments. Liang et al. [67] discovered that poplars absorb pollutants like strontium and diesel oil, while Levei et al. [62] noted that *P. nigra* leaves biomonitor air pollution by accumulating metals from particulate matter, thereby improving urban air quality.

Our study found that poplars exhibit a high degree of adaptability to various street profiles, such as those planted within paved areas, in compact midrise urban areas, and in industrial zones and suburban roadsides as noticed in sycamore maple in the same city [68]. This implies that the studied poplars exhibit the flexibility and ecological advantages necessary to thrive in diverse urban settings. Their ability to adapt to these diverse conditions allows these species to accumulate xenobiotics, thereby effectively aiding in the remediation of traffic-related pollutants. For instance, *P. alba* grows in areas with moderate traffic flow, absorbing pollutants from vehicles and improving air quality, while *P. nigra* ‘Italica’ is found along busy streets, effectively capturing and neutralizing xenobiotics from vehicular emissions. *P. × euramericana* cl. I-214, planted in industrial zones, thrives in mitigating pollution from heavy traffic and industrial activities, thereby contributing to the overall remediation of urban environments. Additionally, the widespread planting of poplars in urban areas can contribute to enhanced urban aesthetics and the overall well-being of city inhabitants, in cases where specimens are not female and/or highly allergenic. These trees offer numerous ecosystem services, including providing shade, improving air quality, and enhancing the overall quality of urban life. However, the management of leaves in phytoremediation systems is an important aspect to consider. Removing leaves from the site may help reduce local pollutant levels, but it is not fully understood how effective this is in removing pollutants from the environment overall, but it is evident that bigger biomass production has a positive effect on phytoremediation effectiveness. On the other hand, if leaves are left to decompose on-site, the pollutants they contain could either degrade in the soil or potentially be reintroduced into the environment, depending on various conditions. Further research is essential to identify the most effective and environmentally safe methods for managing leaves in phytoremediation systems.

## 3. Materials and Methods

### 3.1. Study Area and Site Descriptions

**Figure 7:**
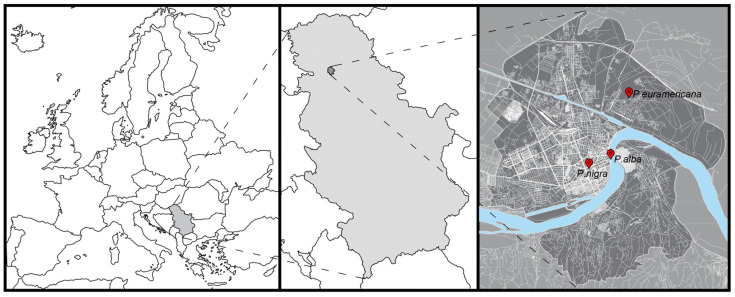
Locations of the analyzed poplar species (*Populus alba, P. × euramericana* cl. I-214, and *P. nigra* ‘Italica’) in Novi Sad (Serbia).

This study examined three locations in Novi Sad, Serbia, Southeast Europe (Figure 7). Novi Sad, situated in the Pannonian plain along the Danube River at an elevation of 76–82 m above sea level, with a high groundwater level ranging from 0.5 to 4 m [69]. The city features a moderate continental climate with warm summers according to the Koppen–Geiger classification [70]. Official climate data from the Republic Hydrometeorological Service of Serbia for the area show an average temperature of 12.2 °C, and the annual sum of precipitation is 675.8 mm over a 30-year period (1995–2024). August is the hottest month (the average minimum temperature is 16.1 °C, and the average maximum temperature is 29.2 °C), while January is the coldest (the average maximum temperature is 4.3 °C, and the average minimum temperature is –2.5 °C). The city faces dry summers, often leading to heat islands, particularly in high-urbanized areas such as streets and squares. The soil found within urban streets, categorized as urbisol, reflects significant human influence and activity.

At each of three studied locations, various species of poplars were observed: white poplar (*Populus alba* L.), Lombardy poplar (*Populus nigra* ‘Italica’ Du Roi), and Euro-American hybrid poplar (*Populus × euramericana* (Dode) Guinier cl. I-214). These species are the most common poplars in urban areas in Novi Sad. For this research, healthy, mature trees in good health condition and characteristic shapes were selected. Qualitative visual assessment of trees useful for research in the urban environment was applied, and only those that demonstrated the highest fitness and an undamaged structure were included. All the sampled tree specimens were approximately the same age.

Furthermore, following the methodology developed by Bechtel et al. [71], differences in the types of local climate zones were observed (Table 2), with each site showing three different types: open midrise, compact midrise, and sparsely built. *Populus alba* has grown in a green strip approximately 5 m wide, situated between the bicycle and pedestrian paths, with the Danube River to the immediate east. Traffic on the street flows in both directions, with two lanes for each direction. On the western side of the street, there are buildings averaging around 20 m in height, in front of which there is a parking space. Dimitrija Tucovića street features a single alley of *P. nigra* ‘Italica’ trees growing between parking slots along one side of the street, characterized by residential buildings ~20 m in height, while on the other side of the street is the city stadium. Additionally, Dimitrija Tucovića is a two-way street, allowing traffic in both directions. *P. × euramericana* cl. I-214 specimens are planted in a copse along the road leading to the suburban settlement of Šangaj, within an industrial zone next to the road.

### 3.2. Data Collection and Preparation

Leaves and one-year-old branches were collected in September 2023 during sunny and dry weather conditions. Approximately 100 g of fresh plant material was collected from the sunny side of the tree canopy. Only undamaged and healthy leaves that were exposed to light were selected for further processing. The collected plant material was placed in paper bags and transported to the laboratory in a portable freezer filled with dry ice. Upon arrival, the leaves and branches were separated and stored at −20 °C. Subsequently, the plant material was frozen in liquid nitrogen and underwent lyophilization for 24 h at −50 °C by using a Martin Christ lyophilizer (Model X, Germany) and was then homogenized using a Retsch ball mill with still cells (mm 400, Germany). The powdered wood and leaf samples were stored in a dark area at room temperature until extraction.

### 3.3. Standards and Reagents

All organic solvents used for extraction such as hexane (HEX), dichloromethane (DCM), acetonitrile, 2-propanol, and methanol were Thermo Fisher GC grade, and were sourced from J.T. Baker (Phillipsburg, NJ, USA). Nitric acid and hydrogen peroxide used for digestion for metal residue analyses were purchased from Thermo Fisher (Waltham, MA, USA). All 10 PCBs’ and 15 PAHs’ external standards, as well as the five surrogate standards for PAH analyses, were GC grade and obtained from Supelco (Bellefonte, PA, USA), with purities exceeding 99%. External standards for heavy metals were acquired from Agilent Technologies (Santa Clara, CA, USA). The surrogate standards consisted of five isotopically labeled PAHs (d_8_-naphthalene, d_10_-acenaphthene, d_10_-phenanthrene, d_12_-chrysene, and d_12_-perylene) at 1 ng μL^−1^ in DCM. The external PAH standards included naphthalene, acenaphthylene, acenaphthene, fluorene, phenanthrene, anthracene, fluoranthene, pyrene, chrysene, benzo(b)fluoranthene, benzo(k)fluoranthene, benzo(a)pyrene, indeno(1,2,3-cd)pyrene, dibenzo(a,h)anthracene, and benzo(g,h,i)perylene. The external PCB standards comprised PCB 8, PCB 20, PCB 28, PCB 35, PCB 52, PCB 101, PCB 118, PCB 153, PCB 138, and PCB 180. Solid phase extraction (SPE) C_18_ cartridges were sourced from Macherey-Nagel (Düren, Germany).

### 3.4. Instrumental Analysis

#### 3.4.1. PAH and PCB Quantification: Extraction and Clean-Up Procedures and GC/MS Analysis

Samples containing 3 g of lyophilized and powdered wood or leaves were spiked with 100 μL of isotopically labeled surrogate standards (c = 100 μg L^−1^) prior to extraction. A microwave-assisted extraction system, Ethos X (Milestone, Italy), was used for PAH and PCB extractions. The 3 g of leaf or wood samples and 40 mL of DCM were placed in 100 mL glass tubes for extraction. The extraction was performed in closed PTFE vessels with a temperature ramp from 40 °C to 110 °C for 20 min and hold at 110 °C for 20 min. The entire extraction process took 40 min.

After extraction, a 25 mL top aliquot was filtered using PTFE syringe filters with a pore size of 0.22 μm and cleaned up with C_18_ SPE cartridges (6 mL, 500 mg absorbent). The SPE cartridges were preconditioned with DCM, 2-propanol, and methanol. Following silica activation, the filtered extracts were applied at a slow flow rate of 1–2 drops per second. Methanol and 2-propanol were used to wash out the SPE cartridges and remove predominantly polar compounds from the extract. Finally, the targeted nonpolar compounds were (1) eluted from the cartridges using DCM, DCM:HEX (1:1), and HEX into glass test tubes; (2) evaporated to dryness using a vacuum concentrator (Martin Christ, RVC 2-18 CDplus); (3) resuspended in DCM; and (4) transferred to GC vials within 200 μL glass inserts.

All PAH and PCB analyses were processed using an Agilent gas chromatograph (GC) model 5975C coupled with a mass spectrometer (MS) 7890 system (Agilent Technologies Inc., Santa Clara, CA, USA) with HP-5MS column (30 m × 0.25 mm × 0.25 µm). A 1 μL sample was injected in spitless mode, following the adapted Agilent application protocol [72]. The temperature ramp was 20 °C min^−1^ up to 230 °C and 10 °C min^−1^ to 315 °C. Finally, the temperature was held at 315 °C for 5 min. The whole run was 25.25 min. A detailed explanation of GC–MS instrumentations, conditions, and method setup as well as quality control procedure was made in detail in the paper of Kostić et al. [47], while analyzed compounds’ retention times, targeted ions, limit of detection (LOD), and limit of quantification (LOQ) values are listed in the Supplementary Material (Table S1).

#### 3.4.2. Heavy Metal: Digestion Procedure and AAS Measurements

A 300 mg sample of freeze-dried and powdered plant material (leaf and wood) was digested using a microwave-assisted digestion system, D series (Milestone, Italy). The digestion process utilized 10 mL of nitric acid, 2 mL of 30% hydrogen peroxide, and 25 mL of deionized water. The digested samples were filtered and analyzed using an Atomic Absorption Spectrometer (model: FS AAS240/GTA120, Varian, CA, USA). For heavy metal quantification, a five-point calibration was employed. An air burner with acetylene as the fuel was used, with an atomization temperature of 2300 °C. All heavy metal analyses were conducted in five biological replicates and two analytical replicates.

### 3.5. Statistical Analyzes

Descriptive statistics were explained with mean values with standard deviation. Two-way analysis of variance (ANOVA) was used with species and organs as variables to identify significant differences among the analyzed samples. The results were interpreted using Fisher’s (F) test and a statistically significance level of 95% (*p* < 0.05). Quantities of accumulated xenobiotics were visually represented using bar charts with Tukey’s HSD post hoc test. Additionally, dendrograms were generated for each type of xenobiotic (PAH, PCB, and HM), with the first and second principal component groups applied in principal component analysis (PCA) for both leaf and branch samples for all three groups of xenobiotics. All data were processed in an R environment [73] using the ‘*ggplot2*’ package [74] and ‘*ggfortify*’ [75] for the visual interpretation of the data. Meanwhile, ANOVA and descriptive statistics were calculated using the ‘*rstatix*’ R package [76].

## 4. Conclusions

All the analyzed poplar species showed high potential for the phytoremediation of PAHs, PCBs, and HMs, as indicated by the detected levels in their tissues. Based on the findings of this study, urban decision makers are encouraged to select poplar species suited to the specific pollutants predominant in a given area, using their ability to accumulate and degrade various xenobiotics. For instance, *P. × euramericana* cl. I-214 demonstrates high potential for the remediation of PAHs and PCBs, making it an ideal choice for areas with significant industrial or vehicular emissions. Likewise, although *P. alba* exhibits slightly lower overall potential for xenobiotic accumulation, it notably accumulates the most CPAHs, emphasizing its importance in remediation strategies related to human health. These findings indicate that the choice of poplar species can be adapted to target specific pollutants more effectively.

*P. nigra* ‘Italica’ demonstrates superior capacity for the accumulation of heavy metals, particularly *Zn*, and could be prioritized in locations with elevated concentrations of heavy metal contaminants. Although *P. alba* exhibits slightly lower overall accumulation potential, its notable ability to absorb carcinogenic PAHs highlights its importance in urban zones with high health risk factors. By combining these species-specific capabilities into urban planning strategies, urban planners and environmentalists can optimize phytoremediation efforts, effectively mitigating pollution from industrial activities, traffic emissions, and other urban sources. This not only contributes to the reduction in environmental contamination but also enhances ecological sustainability, provides valuable ecosystem services, and improves the aesthetic and environmental quality of urban spaces, ultimately promoting a healthier and more sustainable environment for citizens. Additionally, further research should include soil analysis to better understand the interactions between pollutants, plants, and the surrounding environment. Additionally, while poplars have proven to be highly effective for phytoremediation, investigating the potential of other tree species commonly found in urban areas could complement their use. Conducting studies in various urban environments with different types and levels of pollution would help refine species selection and provide more comprehensive ‘*pollution-smart*’ guidelines for integrating phytoremediation strategies into urban planning.

## Figures and Tables

**Table 1:** Results of two-way ANOVA with species (*Populus alba, P. × euramericana* cl. I-214, and *P. nigra* ‘Italica’) and organ (leaf and 1-year-old branch) as dependent variables for all 31 analyzed PAH, PCB, and HM compounds.

Compounds	Abbreviations	Species		Organ		Species × Organ	
		F-Test	*P*	F-Test	*p*	F-Test	*p*
Polycyclic aromatic hydrocarbons (PAHs)							
**Naphthalene**	NAP	8.539	5.00 × 10^−3^	38.616	4.49 × 10^−5^	8.255	6.00 × 10^−3^
**Acenaphthylene**	ACE	1.344	0.297	4.448	0.048	1.149	0.349
**Acenaphthene**	ACT	6.831	1.00 × 10^−2^	44.747	2.23 × 10^−5^	9.113	4.00 × 10^−3^
**Fluorene**	FLU	8.098	0.012	32.680	/	/	/
**Phenanthrene**	PHE	12.780	1.00 × 10^−3^	30.130	1.39 × 10^−4^	22.739	8.28 × 10^−5^
**Anthracene**	ANT	17.227	2.97 × 10^−4^	6.378	0.270	21.239	1.14 × 10^−4^
**Fluoranthene**	FLT	0.375	0.697	2.715	0.130	0.171	0.688
**Pyrene**	PYR	0.828	0.460	6.465	0.026	0.915	0.427
**Chrysene**	CRY	0.832	0.461	2.645	0.132	0.788	0.479
**Benzo[b]fluoranthene**	BbF	0.222	0.804	31.717	1.10 × 10^−4^	0.343	0.716
**Benzo[k]fluoranthene**	BkF	0.469	0.636	16.199	0.002	0.466	0.638
**Benzo[a]pyrene**	BaP	1.139	3.53 × 10^−1^	44.517	2.29 × 10^−5^	0.552	0.591
**Indeno[1,2,3-cd]pyrene**	IcdP	0.765	0.493	0.787	0.398	0.298	0.750
**Dibenzo[a,h]anthracene**	DA	11.456	0.003	16.836	0.002	9.430	0.012
**Benzo[g,h,i]perylene**	BP	1.172	0.346	0.691	0.424	1.057	0.380
**Polychlorinated biphenyls (PCBs)**							
**PCB 8**		6.522	1.20 × 10^−2^	72.674	1.95 × 10^−6^	6.436	1.30 × 10^−2^
**PCB 20**		4.581	0.047	8.200	0.021	3.031	0.105
**PCB 28**		0.891	0.441	0.804	0.391	0.681	0.528
**PCB 35**		19.865	6.35 × 10^−8^	50.271	3.66 × 10^−9^	20.609	5.13 × 10^−8^
**PCB 52**		/	/	/	/	/	/
**PCB 101**		0.787	0.487	0.012	0.914	0.047	0.826
**PCB 118**		0.863	0.454	8.790	0.016	0.710	0.517
**PCB 138**		0.511	0.618	1.253	0.295	0.237	0.795
**PCB 153**		/	/	/	/	/	/
**PCB 180**		36.376	3.85 × 10^−4^	2.025	0.014	/	/
**Heavy metals (HMs)**							
**Cu**		38.201	6.26 × 10^−6^	22.214	5.03 × 10^−4^	6.145	0.015
**Cd**		5.879	0.017	14.960	0.002	4.033	0.046
**Cr**		3.61	0.059	12.701	4.00 × 10^−4^	6.914	0.010
**Fe**		3.914	4.8 × 10^−4^	40.468	3.6 × 10^−5^	2.063	0.170
**Pb**		3.00	0.088	0.119	0.074	0.582	0.574
**Zn**		3.765	0.054	21.209	6.10 × 10^−4^	4.582	0.033
**Mn**		21.822	1.01 × 10^−4^	132.994	7.52 × 10^−8^	6.175	0.014
**Ni**		3.965	0.048	11.616	0.005	1.848	0.200

**Table 2:** Locations with coordinates and urban morphology characteristics such as urbanization level, type of local climate zones, and street canyon characteristics.

Species	Location	Coordinates		Type of Local Climate Zone	Street Orientation	Degree of Urbanization (%)	Mean Street Canyon Width (m)	Mean Street Canyon Height (m)	Height/Width Ratio	Altitude (m a.s.l.)
		**Latitude**	**Longitude**							
*Populus alba*	Sunčani kej	45.251668	19.855855	Open midrise	N-S	30	35.97	32.40	0.90	74.69
*Populus nigra* ‘Italica’	Dimitrija Tucovića	45.247900	19.842148	Compact midrise	NE-SW	56	17.20	15.17	0.88	76.05
*Populus × euramericana* cl. I-214	Šajkaškog odreda	45.280335	19.854746	Sparsely built	E-W	21	25.82	23.20	0.90	75.85

## Data Availability

The original contributions presented in this study are included in the article/Supplementary Materials. Further inquiries can be directed to the corresponding author.

## Supplementary Materials

The following supporting information can be downloaded at https://www.mdpi.com/article/10.3390/plants14101445/s1, Table S1. PAH compounds number of rings, category, retention time, quantifier, qualifier ions, LOD and LOQ values.
