# Supplementary material for: Populus × euramericana Accumulates More Organic Pollutants (PAHs and PCBs), While P. nigra ‘Italica’ Absorbs More Heavy Metals
Source: Plants (Basel). 2025 May 12;14(10):1445. doi: 10.3390/plants14101445 (PMC12115039; doi:10.3390/plants14101445)
Supplement: Supplementary file 1 [file plants-14-01445-s001.zip › plants-3613311-supplementary.pdf]

TITLE:

Populus × euramericana Accumulate More Organic Pollutants (PAHs and PCBs) While *P. nigra* 'Italica' Absorb More Heavy Metals

Table S1. PAH compounds number of rings, category, retention time, quantifier, qualifier ions, LOD and LOQ values.

Compound	Abbreviation	No. of rings	Category *	Retention times (min.)	Quantifier/ Qualifier ions	LOD** (ng g^{-1})	LOQ*** (ng g^{-1})	Linearity (r^2)
**Naphthalene**	NAP	2	L-PAHs	6.071	128/127, 129	0.19	0.58	>0.99
**Naphthalene-d_8**	NAP-d8		/	6.142	136/134, 108	0.02	0.75	>0.99
**Acenaphtylene**	ACE	3	L-PAHs	7.792	152/151, 153	0.40	1.21	>0.99
**Acenaphthene-d_{10}**	ACE-d10	3	L-PAHs	8.154	164/162, 160	0.14	0.43	>0.99
**Acenaphthene**	ACT	3	L-PAHs	8.206	153/154, 152	0.00	0.00	>0.99
**Fluorene**	FLU	3	L-PAHs	8.787	166/165, 163	0.00	0.00	>0.99
**Phenanthrene-d_{10}**	PHE-d10		/	9.932	188/189	0.05	0.22	>0.99
**PCB 8**	PCB 8		/	9.404	221/223, 186	0.02	0.13	>0.99
**Phenanthrene**	PHE	3	L-PAHs	9.961	178/176, 179	0.02	0.06	>0.99
**Anthracene**	ANT	3	L-PAHs	10.019	178/176, 179	0.06	0.18	>0.99
**PCB 20**	PCB 20		/	10.459	255/257, 186	0.03	0.22	>0.99
**PCB 28**	PCB 28		/	10.601	256/258, 186	0.05	0.17	>0.99
**PCB 52**	PCB 52		/	10.869	292/290, 222	0.01	0.13	>0.99
**PCB 35**	PCB 35		/	11.164	257/259, 186	0.04	0.25	>0.99
**Fluoranthene**	FLT	4	M-PAHs	11.951	202/200, 203	0.03	0.10	>0.99
**PCB 101**	PCB 101		/	12.250	326/328, 254	0.03	0.31	>0.99
**Pyrene**	PYR	4	M-PAHs	12.436	202/200, 203	0.03	0.10	>0.99
**PCB 118**	PCB 118		/	13.433	326/328, 254	0.09	0.24	>0.99
**PCB 153**	PCB 153		/	13.995	308/310	0.05	0.33	>0.99
**PCB 138**	PCB 138		/	14.436	360/362, 290	0.04	0.28	>0.99
**Chrysene-d_{12}**	CRY-d12	4	/	15.332	240/236, 241	0.13	0.40	>0.99
**Crysene**	CRY	4	M-PAHs	15.395	228/226, 229	0.38	1.16	>0.99
**PCB 180**	PCB 180			15.901	394/396, 324	0.11	0.74	>0.99

**Benzo(b)fluoranthene**	BbF	5	H-PAHs	17.804	252/250, 126	0.17	0.51	>0.99
**Benzo(k)fluoranthene**	BkF	5	H-PAHs	17.809	252/250, 126	0.00	0.00	>0.99
**Benzo(a)pyrene**	BaP	5	H-PAHs	18.437	252/253, 250	0.20	0.61	>0.99
**Perylene-d12**	PER-d12		/	18.437		0.10	0.45	>0.99
**Ideno(1,2,3-cd)pyrene**	IcdP	6	H-PAHs	20.662	276/277, 274	0.16	0.48	0.98
**Dibenzo(a,h)anthracene**	DA	5	H-PAHs	20.960	278/276, 279	0.15	0.46	>0.99
**Benzo(g,h,i)perylene**	BP	6	H-PAHs	21.118	276/277, 274	0.03	0.10	0.98

(*) L-PAHs-low-weight PAHs; M-PAHs-Medium-weight PAHs; H-PAHs-Heavy-weight PAHs; (**) LOD-Limit of Detection;

(***) LOQ-Limit of Quantification.
